# Broken Heart Syndrome: A Rare Case of Recurrent Reverse Takotsubo Cardiomyopathy Associated with Acute Respiratory Failure

**DOI:** 10.31661/gmj.v12i.3103

**Published:** 2023-12-01

**Authors:** Samina Jabeen, Deepak J Pattanshetty

**Affiliations:** ^1^ Division of Internal Medicine, Primary Health Care Corporation Doha, Qatar; ^2^ Division of Cardiology, Pikeville Medical Center, Kentucky, USA

**Keywords:** Reverse Takotsubo Cardiomyopathy, Stress Induced Cardiomyopathy, Nonischemic Cardiomyopathy, Inverted Takotsubo Cardiomyopathy

## Abstract

Background: Takotsubo cardiomyopathy accounts for one percent of acute coronary syndrome presentations and has been increasingly recognized [1]. Reverse Takotsubo cardiomyopathy, a variant form of Takotsubo cardiomyopathy presenting with the hyperdynamic function of the apical segments and hypokinesis of the basal or mid-ventricular segments is the rarest type of acute stress cardiomyopathy, with mid-ventricular akinesia and preservation of apical and basal contractility [2]. Case Report: We report a rare case of an elderly woman admitted to the Intensive Care Unit at Case Western Reserve University Hospital in Cleveland, USA. The patient experienced acute respiratory failure as a result of exacerbated chronic obstructive lung disease and heart failure. Echocardiography revealed reverse Takotsubo cardiomyopathy. Cardiac catheterization showed nonobstructive coronary artery disease. The wall motion abnormalities resolved within two weeks. The case is unique in that she had an identical presentation one year earlier after she had entered the same dusty room! Conclusion: Our case report is unique and describes the rarest form of recurrent reverse Takotsubo cardiomyopathy. Our case demonstrates that reverse Takotsubo cardiomyopathy with identical wall motion abnormalities can recur in a patient upon re-exposure to a similar stressful situation. Early recognition and appropriate treatment can prevent catastrophic outcomes.

## Introduction

Takotsubo cardiomyopathy, also known as "stress cardiomyopathy" or "broken heart syndrome," was first described in 1983 in Japanese women [1]. It predominantly occurs in postmenopausal women and is usually triggered by emotional or physical stress or by a critical illness. It is an acute but often reversible left ventricular (LV) dysfunction. Takotsubo cardiomyopathy has a clinical presentation often resembling acute coronary syndrome (ACS) without evident obstructive coronary artery disease on angiogram, characterized by a transient apical akinesis/hypokinesis and basal hyperkinesis of the heart. Usually, Takotsubo cardiomyopathy resolves spontaneously in a few days or weeks. There are variant forms of Takotsubo cardiomyopathy including reverse Takotsubo cardiomyopathy that present with different patterns of ventricular systolic dysfunction [2]. Reverse Takotsubo cardiomyopathy is the rarest type of acute stress cardiomyopathy, with mid-ventricular akinesia and preservation of apical and basal contractility [3]. Here, we report the rare case of an elderly woman admitted to our intensive care unit (ICU) with acute respiratory failure following an inhalation of dust from her attic. Echocardiography revealed reverse Takotsubo cardiomyopathy. Cardiac catheterization showed mild diffuse coronary artery disease. The wall motion abnormalities resolved within two weeks. The case is unique in that she had an identical presentation one year earlier after she had entered the same dusty room!

Case Presentation

An 81-year-old elderly woman with chronic obstructive lung disease (COPD), hypertension, and diabetes mellitus was admitted to the Casewestern Reserve University Hospital ICU in Cleveland, USA, with acute respiratory failure due to COPD exacerbation after exposure to dust. She was treated with non-invasive ventilation, steroids, and bronchodilators. As part of the evaluation of dyspnea, serum troponin I levels were measured and found elevated with a peak of 1.7 mg/dL. An electrocardiogram showed sinus tachycardia, left axis deviation, and poor R wave progression in anterior leads. The rest of her laboratory tests were unremarkable.

**Figure-1:**
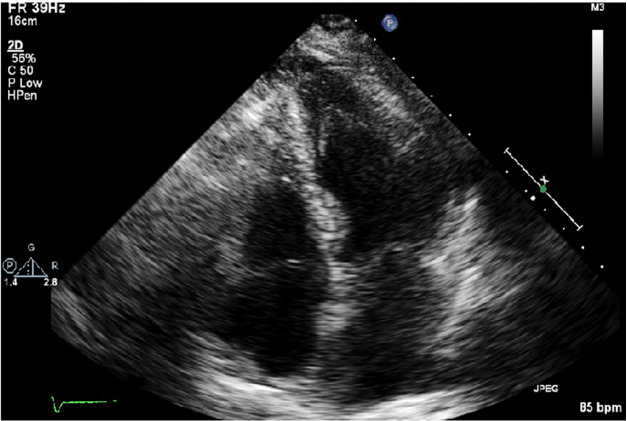
Figure-1. Apical 4 chamber view showing hypokinetic mid-septal and mid-lateral walls and a normal apex and base.

**Figure-2:**
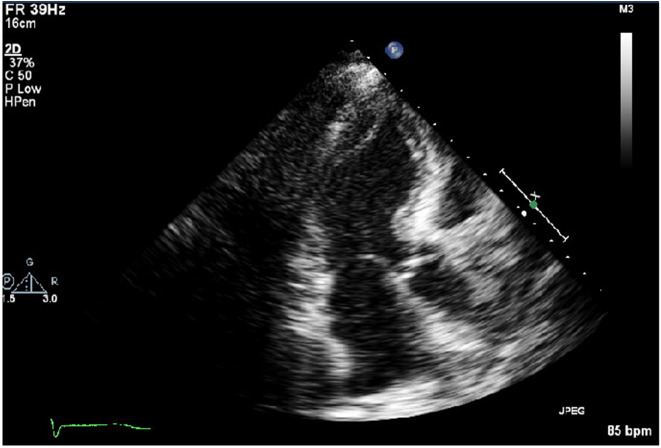
Figure-2. Apical long axis view showing hypokinetic mid-inferolateral and mid-septal walls and normal apex and base

**Figure-3:**
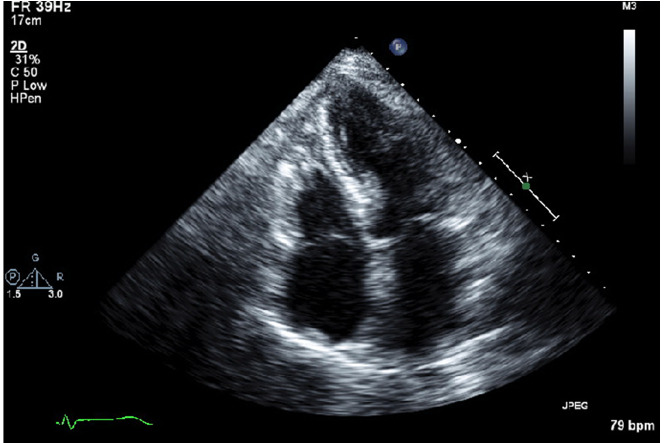
Figure-3. Apical 4 chamber view showing a normal function of the mid-septal and mid-lateral walls

A transthoracic echocardiogram (Figure-1, -2) demonstrated a reduced left ventricular ejection fraction (35%) with hypokinesis of mid-anteroseptal, mid-lateral, mid-inferior, and mid-anterior walls with the preserved function of both base and apex of the heart; these findings suggested Reverse Takotsubo cardiomyopathy. Subsequently, a left heart catheterization was performed which showed mild diffuse coronary artery disease. A ventriculogram confirmed the echocardiographic findings. The patient was treated with a beta blocker, angiotensin-converting enzyme inhibitor, diuretics, and aspirin. A follow-up echocardiogram two weeks later showed complete normalization of her left ventricular function (Figure-3).

A unique aspect of the case was having an identical presentation with identical wall motion abnormalities the previous year after exposure to the same dusty attic. Her echocardiogram at the time also showed reverse Takotsubo cardiomyopathy and a repeat echocardiogram two weeks later had normalized.

## Discussion

Takotsubo cardiomyopathy accounts for one percent of acute coronary syndrome presentations and has been increasingly recognized [4]. Recently, a variant form of Takotsubo cardiomyopathy has been described presenting with hyperdynamic function of the apical segments and hypokinesis of the basal or mid-ventricular segments, hence called ‘inverted’ or ‘reverse cardiomyopathy’ [2].

Here we present a rare case of reverse or intervened Takotsubo cardiomyopathy. The patient was exposed to attic dust which caused exacerbation of chronic obstructive lung disease leading to acute respiratory failure. This in-turn precipitated congestive heart failure and an echocardiogram showed hypokinesis of mid-anteroseptal, mid-lateral, mid-inferior, and mid-anterior walls with the preserved function of both base and apex of the heart, which was characteristic of reverse Takotsubo cardiomyopathy.

The pathophysiology of this variant of Takotsubo cardiomyopathy is uncertain but our observation that a recurrent episode exhibited identical wall motion changes is consistent with the hypothesis that a sympathetic surge is the trigger; the locations of the wall motion abnormalities are then governed by the specific distribution of the sympathetic innervations of the heart [4].

The clinical features of reverse Takotsubo cardiomyopathy are similar to classic Takotsubo cardiomyopathy. The common clinical presentation is often chest pain, indistinguishable from acute coronary syndrome. Other presentations include dyspnea, syncope, shock, and electrocardiographic abnormalities [4.5]. Post-menopausal women are frequently affected as reported in other stress cardiomyopathies [4]. The Mayo Clinic has suggested four very useful criteria to confirm the diagnosis of Takotsubo cardiomyopathy: (1) transient hypokinesis, akinesis, or dyskinesis of the left ventricular mid segments with or without apical involvement with the regional wall motion abnormalities extending beyond a single epicardial vascular distribution, (2) absence of obstructive coronary disease or angiographic evidence of acute plaque rupture, (3) new ECG abnormalities or elevation in cardiac troponin, and (4) absence of pheochromocytoma and myocarditis [6].

Reverse Takotsubo cardiomyopathy is usually transient in nature and ventricular dysfunction resolves in about 1-4 weeks. Hence treatment remains standard supportive care for congestive heart failure. Beta-blocker therapy has been suggested for long-term protection against catecholamine surges. The patient was treated aggressively with bronchodilators, b-blockers, angiotensin inhibitors, and diuretics, following which her symptoms resolved. We followed her in our clinic after 2 weeks, the echocardiogram was repeated and all her wall motion abnormalities were resolved.

The reoccurrence of reverse Takotsubo cardiomyopathy is considered rare. Gianni et al .[7] identified 4 studies documenting a mean recurrence rate of 3.5% in classical Takotsubo cardiomyopathy but the recurrence rate in the reverse pattern is unknown. One year ago, the patient had a similar presentation of acute respiratory failure and congestive heart failure after exposure to attic dust. Her echocardiogram at the time also showed reverse Takotsubo cardiomyopathy and a repeat echocardiogram two weeks later had normalized.

## Conclusion

This report demonstrates a rare case of reverse or inverted Takotsubo cardiomyopathy which was associated with acute respiratory failure. Unique and a very rare aspect of our case report is that she had an identical presentation with similar wall motion abnormalities, the prior year after exposure to the same dusty attic. Our case demonstrated that reverse Takotsubo cardiomyopathy with identical wall motion abnormalities can recur when re-exposed to similar stressful situations. Hence early recognition and appropriate management can prevent catastrophic outcomes.

## Conflict of Interest

There is no conflict of interest.
